# Supplementary material for: Experimental certification of millions of genuinely entangled atoms in a solid
Source: Nat Commun. 2017 Oct 13;8:907. doi: 10.1038/s41467-017-00898-6 (PMC5640624; doi:10.1038/s41467-017-00898-6)
Supplement: Supplementary file 1 — Supplementary Information [file 41467_2017_898_MOESM1_ESM.pdf]

Supplementary Information for: “Experimental certification of millions of genuinely entangled atoms in a solid”

Supplementary Note 1 - Fluorescence measurement

The measurement of the incoherent reemission (the fluorescence) in the backward $\mathbf{k}_\mathrm{b}$ and the forward $\mathbf{k}_\mathrm{f}$ modes shown on Fig. 1 (b) in the main text required several corrections. First, the coupling efficiencies were measured independently to be equal to more than 70% inside the optical fibre. A slight imbalance in the couplings was taken into account. Another correction was necessary due to the non-perfect transmission of the narrow bandpass filter (FWHM of 10 nm at 883.2 nm wavelength) that was used in the backward mode. The transmission was measured to be 65% at the wavelength of the heralded single photon. Finally, another correction is attributed to the PBS used in the backward mode. To apply it the polarization state of the incoherent re-emission has to be characterized. Due to the use of polarization preserving quantum memory (consisting of two crystals separated by the half-wave plate [1]) and a bow-tie configuration, the polarization state of the fluorescence emitted in backward and forward is close to a mixed state. To confirm it we performed a polarization state tomography of the emitted fluorescence. We obtained the purity of 51% and the fidelity of 95% with respect to the completely mixed polarization state. Based on this result a correction of 0.5 was used in all measurements.

In a single crystal, the absorption probability for every spatial position inside the crystal is not the same. Hence, the total probabilities to have incoherent reemission (the fluorescence) in the forward or backward directions are not equal. Assuming the total optical depth of the crystal is d one can write the ratio J between emission rate between two modes as

$$J = \frac{2de^{-d}}{1 - e^{-2d}}. \tag{1}$$

However, due to the use of the double-pass configuration in our experiment, this asymmetry disappears. More precisely, the amount of the emitted fluorescence in forward and backward modes should be equal. This was verified by the direct and simultaneous measurement of the fluorescence in both the forward and backward modes. We found a ratio of at most $J = 1.03(10)$ over a time interval of more than 800 µs after the absorption of the optical pulse, as shown in Supplementary Figure 1 (a).

To measure the SNR the strong coherent state pulses were coupled to the QM prepared in the crystal. Generally, the SNR is fundamentally limited by the number of atoms N involved to the collective re-emission process and can be expressed as

$$\mathrm{SNR} = \frac{\eta\,|\alpha|^2}{|\alpha|^2/N + \delta}, \tag{2}$$

where $|\alpha|^2$ is the absorbed mean photon number of the coherent state pulse, η is the rephasing efficiency of the QM and δ is the contribution from the intrinsic noise of the detection system. This expression is correct for a single coherent state pulse or in the limit of the low repetition rate R of the experiment ($R \ll 1/T_1$, T_1 being the spin relaxation time). In the general case, taking into account accumulation of the population in the excited state which comes from many coherent state pulses one can write SNR as

$$\mathrm{SNR} = \frac{\eta\,|\alpha|^2}{|\alpha|^2/(N(1 - e^{-1/RT_1})) + \delta}. \tag{3}$$

This expression can be used to verify the source of the fluorescence and finally to estimate the number of atoms that are contributing to the collective emission. First, for the fixed repetition rate of $R = 1$ MHz, the input intensity of the coherent state pulses was varied to see the influence of the noise from the detection system δ (Supplementary Figure 1 (b)). The maximum measured SNR value was 72 dB for the high mean photon number at the input where the detector's noise contribution is negligible. While decreasing $|\alpha|^2$ its contribution starts to be more dominant which decreases SNR. The model based on Supplementary Equation (3) and the independently measured parameters explains well the obtained data.

Next, the repetition rate R was varied for the fixed $|\alpha|^2$ which is high enough to make the detector's noise contribution negligible (Supplementary Figure1(c)). In this case higher values of the SNR can be obtained due to the lower accumulation of the population in the excited state (such that $R \ll 1/T_1$ which corresponds to the highest SNR value). The fluorescence lifetime T_1 was measured to be 250 µs from separate measurement. The only free parameter to fit the data is the number of atoms N, which was estimated to be 106.0(1) dB $\approx 4.0(1) \times 10^{10}$ atoms. The maximal SNR for the measurement with the heralded single photon is expected to be higher since the spectral bandwidth of the AFC structure in this case is five times larger. This means that the number of atoms that contribute to the absorption of a single photon is larger than the measured N. Nevertheless, we do not correct the measured SNR but directly work with N.

Supplementary Note 2 - Simplification of the minimization problem

Equation (9) in the main text is a constrained minimization problem over $3M$ complex numbers. Here, we show how to reduce the complexity with a few simple arguments.

First, note that state Eq. (6) in the main text could be subnormalized, because we neglect populations in other subspaces. However, it is straightforward to see that subnormalized states give the same (p_1, p_2) values as the renormalized state mixed with the ground state. Hence, it is sufficient to consider normalized states only.

Second, we choose $a_i \geq 0$ without loss of generality. For the phases of the b_i and c_i, note that the b_i dependent term in Eq. (8) in the main text can be written as

$$\sum_{i<j} \frac{b_i b_j}{a_i a_j} = \frac{1}{2}\left(\sum_i \frac{b_i}{a_i}\right)^2 - \frac{1}{2}\sum_i \left(\frac{b_i}{a_i}\right)^2. \tag{4}$$

Let us write $b_i = e^{i\varphi_i}|b_i|$ and $\sum_i b_i/a_i = e^{i\bar{\varphi}}|\sum_i b_i/a_i|$. Then, with $c_i = e^{i\vartheta_i}|c_i|$, Eq. (8) in the main text reads

$$\begin{aligned} p_2 = \frac{|A|^2}{M^2} \Bigg| \frac{1}{\sqrt{2}} \left|\sum_i \frac{b_i}{a_i}\right|^2 \\ - \frac{1}{\sqrt{2}} \sum_i e^{2i(\varphi_i - \bar{\varphi})} \left|\frac{b_i}{a_i}\right|^2 + \sum_i e^{i(\vartheta_i - 2\bar{\varphi})} \left|\frac{c_i}{a_i}\right| \Bigg|^2 . \end{aligned} \tag{5}$$

Clearly, the phases have to be set to $\varphi_i = \bar{\varphi}$ and $\vartheta_i = \pi + 2\bar{\varphi}$ in order to minimize p_2. Without loss of generality, we set $\bar{\varphi} = 0$ implying that $b_i \in \mathbb{R}$ and $c_i \leq 0$. Note that the case where $\sum_{i<j} \frac{b_i b_j}{a_i a_j} < 0$ is not interesting here because it is only possible

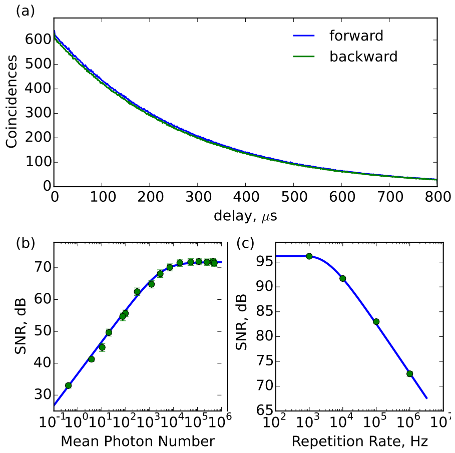

Supplementary Figure 1. Signal-to-noise ratio (SNR) measurement using strong coherent states. (a) The fluorescence lifetime measurement in forward and backward spatial modes. A lifetime of the excited state of $T_1 = 250$ μs was obtained from the exponential fit. Both curves overlap almost perfectly up to at least 800 μs after the absorption of the strong coherent pulse. The coherent emission in the forward mode at 50 ns is not shown. (b) The measured SNR in backward mode using strong coherent state pulses as a function of mean photon number per pulse $|\alpha|^2$. Due to the dominated contribution from the detector's noise δ for low $|\alpha|^2$ the SNR value goes down. The solid line is a model curve based on Supplementary Equation (3) and independently measured parameters (with N as a free parameter). The repetition rate was fixed to 1 MHz. (c) SNR as a function of the repetition rate of the pulses for $|\alpha|^2 = 10^6$. The solid line is a fit based on the Supplementary Equation (3) where the number of atoms N is the only free parameter. The estimation gives at least $N = 106.0(1)$ dB which agrees well with the value obtained from the doping concentration. All error bars represent one standard deviation of the measured uncertainty.

for so-called subradiant states, that is, states with lower intensity in forward direction than the incoherent emission.

To summarize, we have $a_i \geq 0$, $b_i \in \mathbb{R}$ and $c_i = -\sqrt{1 - a_i^2 - b_i^2}$, thus reducing the problem to $2M$ real parameters. The simplified formulas read

$$p_1 = \frac{A^2}{M} \left(\sum_i \frac{b_i}{a_i} \right)^2 \tag{6}$$

and

$$p_2 = \frac{A^2}{M^2} \left(\sqrt{2} \sum_{i<j} \frac{b_i b_j}{a_i a_j} + \sum_i \frac{c_i}{a_i} \right)^2 . \tag{7}$$

Supplementary Note 3 - Formulas from the Lagrange multiplier

The partial derivatives of Eq. (10) in the main text are

$$\frac{\partial f}{\partial a_i} = \frac{f + \lambda C}{a_i} - \frac{A}{a_i}\left(\frac{\sqrt{2}b_i}{a_i}\sum_{j\neq i}\frac{b_j}{a_j} + \frac{a_i}{c_i} + \frac{c_i}{a_i} + \lambda\frac{b_i}{a_i}\right), \tag{8}$$

$$\frac{\partial f}{\partial b_i} = \frac{A}{a_i}\left(\sqrt{2}\sum_{j\neq i}\frac{b_j}{a_j} - \frac{b_i}{c_i} + \lambda\right) \tag{9}$$

and

$$\frac{\partial f}{\partial \lambda} = f_1 - C. \tag{10}$$

From $\partial f/\partial b_i = 0$, we find

$$\frac{b_i}{c_i} = \sqrt{2}\sum_{j\neq i}\frac{b_j}{a_j} + \lambda, \tag{11}$$

which we insert into $\partial f/\partial a_i = 0$ and find

$$\frac{1}{a_i c_i} = \frac{f + \lambda C}{A}. \tag{12}$$

Supplementary Equation (11) can also be written as

$$b_i\left(\frac{1}{c_i} + \sqrt{2}\frac{1}{a_i}\right) = \sqrt{2}\sum_{j}\frac{b_j}{a_j} + \lambda. \tag{13}$$

We notice that from $\partial f/\partial a_i = 0$ and $\partial f/\partial b_i = 0$ we find Supplementary Equations (12) and (13), where the right hand side for both is independent of i. Hence, it follows that

$$a_i c_i = a_j c_j \tag{14}$$

and

$$b_i\left(\frac{1}{c_i} + \frac{\sqrt{2}}{a_i}\right) = b_j\left(\frac{1}{c_j} + \frac{\sqrt{2}}{a_j}\right) \tag{15}$$

for all pairs (i, j). Let us fix $j = 1$ and write $a \equiv a_1$, $b \equiv b_1$ and $c \equiv c_1$. Supplementary Equations (14) and (15) thus give two equations for two unknowns (a_i, b_i) (recall that c_i is just a function of a_i, b_i).

Inserting Supplementary Equation (14) into Supplementary Equation (15) allows to eliminate b_i. The remaining equation is a polynomial of degree four in a_i^2. One

finds the four solutions

$$a_i = \begin{cases} a \\ \sqrt{\frac{x_0}{3}\left(1+(-1)^{2/3}\frac{x_1}{x_2}+(-1)^{-2/3}x_2\right)} \\ \sqrt{\frac{x_0}{3}\left(1+\frac{x_1}{x_2}+x_2\right)} \\ \sqrt{\frac{x_0}{3}\left(1+(-1)^{-2/3}\frac{x_1}{x_2}+(-1)^{2/3}x_2\right)}, \end{cases} \tag{16}$$

where

$$x_0 = 1-a^2-2\sqrt{2}ac, \tag{17}$$

$$x_1 = 1-6c^2\frac{1-c^2-\sqrt{2}ac}{x_0^2} \tag{18}$$

and

$$\begin{aligned} x_2 = \Bigg\{ 1 - \frac{3\sqrt{3}c^2|b|}{x_0^3} \Big[& 2a^2(1-a^2)^2+8c^2(1-c^2)^2 \\ & + 2\sqrt{2}ac\left(6a^4+12c^4-14c^2+3+24a^2c^2-7a^2\right) \\ & + a^2\left(48c^4-36c^2+48a^2c^2\right)-b^2\Big]^{1/2} \\ & - \frac{9c^2}{x_0^3}\left(2a^2c^2+\sqrt{2}ac\left(a^2+2c^2-3\right)+b^2\right)\Bigg\}^{1/3}. \end{aligned} \tag{19}$$

The solutions for b_i and c_i follow accordingly.

Supplementary Note 4 - Asymptotic formula for p_1

To argue that for large M the only relevant configuration is the symmetric one, we calculate p_1 for arbitrary configurations and show that all but the symmetric configuration are asymptotically outside the relevant interval I (see Methods). To this end, note the factor $A^2 = \Pi_{i=1}^{M} a_i^2$ in the Supplementary Equations (6) and (7). For large M, this implies that almost all a_i have to be very close to one. However, solutions two to four in Supplementary Equation (16) are close to zero for a close to one. Asymptotically, we expect that $a_i = 1 - O(1/M)$ to have a finite A. To see this, consider $b = \beta/\sqrt{M}$ and $c = -\gamma/\sqrt{M}$ with $\beta, \gamma = O(1)$ and do a Taylor series of the solutions Supplementary Equation (16) around $1/M = 0$. One finds

$$a_i = \begin{cases} \sqrt{1-(\beta^2+\gamma^2)/M} \\ \sqrt{\gamma}(2M)^{-1/4} - \sqrt{\frac{\beta}{M}} + O(M^{-3/4}) \\ \sqrt{\gamma}(2M)^{-1/4} + \sqrt{\frac{\beta}{M}} + O(M^{-3/4}) \\ \frac{\gamma}{\sqrt{M}} + O(M^{-2}) \end{cases} \tag{20}$$

and

$$b_i = \begin{cases} \dfrac{\beta}{\sqrt{M}} \\ 1 - \dfrac{3\gamma}{2\sqrt{2M}} - \dfrac{\beta\sqrt{\gamma}}{2(2M)^{3/4}} + O(M^{-1}) \\ 1 - \dfrac{3\gamma}{2\sqrt{2M}} + \dfrac{\beta\sqrt{\gamma}}{2(2M)^{3/4}} + O(M^{-1}) \\ \dfrac{\beta}{\sqrt{M}} + O(M^{-3/2}) \end{cases} \tag{21}$$

We therefore see that only configurations with $\delta m = \sum_{j=2}^{4} m_j = O(1)$ asymptotically give finite values of A. More explicitly, inserting Supplementary Equations (20) and (21) into p_1 for a fixed configuration C with $\delta m = O(1)$ gives

$$p_1 = M^{-\frac{1}{2}(m_2+m_3+2m_4)} \left(2^{\frac{1}{2}(m_2+m_3)} e^{-\beta^2-\gamma^2} \beta^2 \gamma^{m_2+m_3+2m_4} + O(\frac{1}{M^{\frac{1}{4}}})\right). \tag{22}$$

A simple calculation shows that the maximum of Supplementary Equation (22) over all β, γ and $\delta m > 0$ is $p_1^{\max} = (e^3 M)^{-1/2} + O(1/M)$. This means that for large enough M, all but the symmetric configuration exhibit a $p_1^{\max}$ that do not enter the nontrivial interval I (which has a lower bound $(eM)^{-1/2} + O(1/M)$).

Supplementary Note 5 - Example $M = 3$

We discuss an example where a nonsymmetric solution partially constitutes the global minimum. This is because for $M = 2, 3, 4$, $p_1^{\lim 1} > p_1^{\lim 2}$ (see Methods). For $M = 3$, one has $p_1^{\lim 1} = 1/3$ and $p_1^{\lim 2} \approx 0.29$.

In Supplementary Figure 2, we compare the "full" numerical constrained minimization of Supplementary Equation (7) without the Lagrange multiplier method with a constrained minimization over (a, b) for two relevant configurations C. We see that the kink for the full minimization is nicely explained by the crossing of the minimization for two different configurations.

Supplementary References

[1] Clausen C, Bussières F, Afzelius M and Gisin N, *Phys. Rev. Lett.* **108** 190503 (2012).

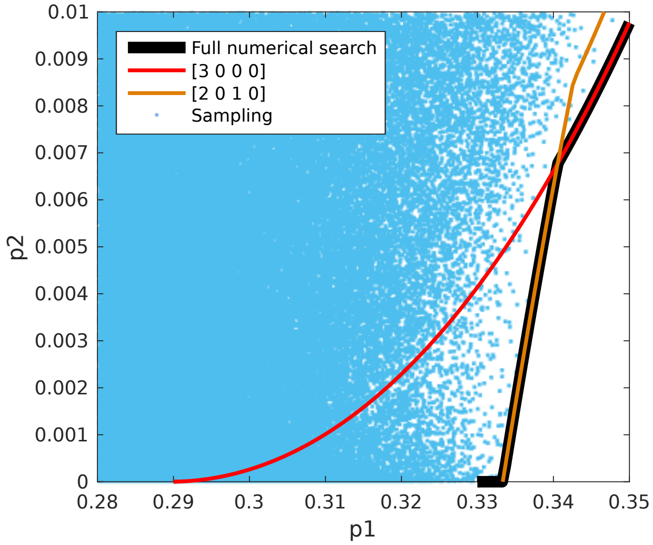

Supplementary Figure 2. Zoom in the (p_1, p_2) plane for $M = 3$. The thick black curve is the full numerical minimization of p_2 given p_1. The two colored, thinner lines are two relevant configurations (red is the symmetric configuration). One clearly identifies $p_1^{\lim 1} = 1/3$ and $p_1^{\lim 2} \approx 0.29$. The full search follows the minimum of the two configurations. Further confidence is gained by sampling millions of states (blue dots).
